# Tetraspanin-enriched microdomains: The building blocks of migrasomes

**DOI:** 10.1016/j.cellin.2021.100003

**Published:** 2022-01-05

**Authors:** Yuwei Huang, Li Yu

**Affiliations:** State Key Laboratory of Membrane Biology, Beijing Frontier Research Center for Biological Structure, School of Life Science, Tsinghua University-Peking University Joint Center for Life Sciences, Tsinghua University, Beijing, China

**Keywords:** Migrasome, Tetraspanins, Microdomains, Tetraspanin-enriched microdomains

## Abstract

The migrasome is a newly discovered organelle of migrating cells. Migrasomes play diverse physiological roles including mitochondrial quality control, lateral transfer of material between cells, and delivery of signaling molecules to spatially defined locations. The formation of migrasomes is dependent on tetraspanins, a group of membrane proteins containing four transmembrane domains, which form membrane microdomains named tetraspanin-enriched microdomains (TEMs). In this review, we will discuss the mechanisms for migrasome biogenesis, with a focus on the role of TEMs and the organizing principles underlying the formation of TEMs.

## 1 Discovery of migrasomes

**Fig. 1:**
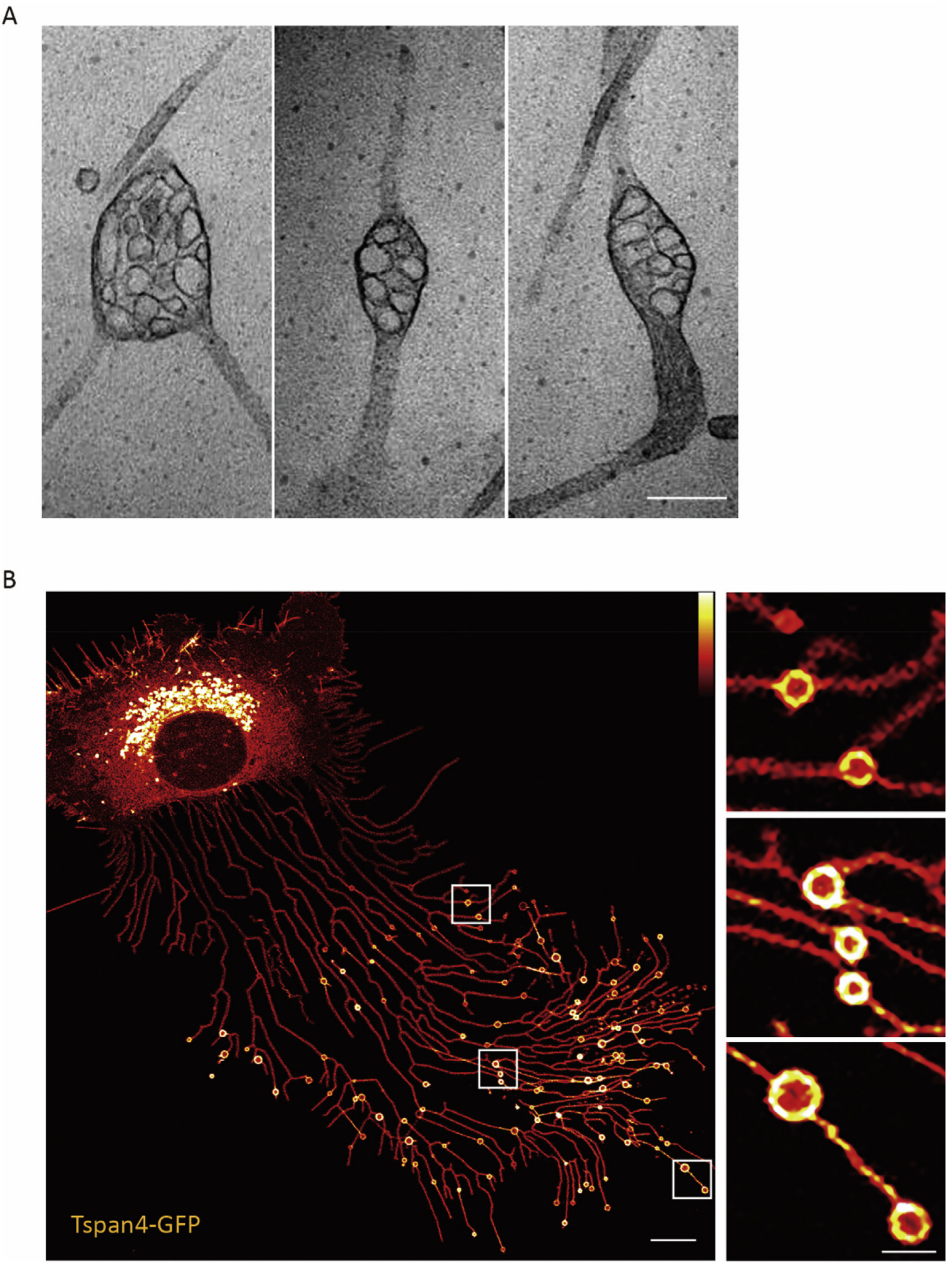
**Discovery of migrasomes** A. Pomegranate-like structures outside of cells observed by transmission electron microscopy. Scale bar, 250 nm. B. Heat-map image of migrasomes and retraction fibers generated by L929 cells overexpressing Tspan 4-GFP. Scale bar, 10 μm; zoom in, 2 μm.

In 2012, while using transmission electron microscopy, we observed large vesicular structures outside cells, which were around 2 μm in size and with numerous intraluminal vesicles (Fig. 1A). Since these structures looked like opened pomegranates, we jokily call them “pomegranate-like structures” (PLSs) in the lab.

To learn more, we purified the pomegranate-like structures and analyzed their protein composition by mass spectrometry. The enriched proteins on PLSs were subjected to imaging-based screening. By this approach, we identified Tetraspanin4 (Tspan4) as a marker to visualize the PLSs by confocal microscopy (Fig. 1B). Live-cell imaging revealed that PLSs grow on retraction fibers, an elaborate network of fine membrane tethers on the trailing edge of a migrating cell. Very soon, we learned that formation of PLSs actually depends on cell migration. Through correlative confocal and transmission electron microscopy analysis, we confirmed that the vesicles observed in the confocal images were indeed the pomegranate-like structures observed by the transmission electron microscope. Since formation of PLSs is migration dependent, we formally named them as migrasomes (Ma et al., 2015).

Since those initial discoveries, Tspan4 and the pleckstrin homology (PH) domain fused to mCherry or GFP have been used to visualized migrasomes for live-cell imaging. In addition, we found that wheat germ agglutinin (WGA) stains migrasomes well. An easy-to-use protocol for migrasome staining has now been developed for cultured cells (Chen et al., 2019). Further analysis using these probes revealed that migrasomes are formed on the branch points or the tips of retraction fibers, and they grow up to 0.5–3 μm in diameter. The life time of migrasomes in cultured cells is around 4 h before they rupture or become engulfed by other cells. Migrasomes have been observed in different cell lines including MEF, NIH3T3, L929, HaCaT, MGC803, B16 and MDA-MB-231, and in primary cells including mouse embryonic stem cells, mouse hippocampal neurons, mouse bone marrow-derived macrophages, human podocytes and neutrophils (Ma et al., 2015; Liu et al., 2020; Jiao et al., 2021). Importantly, migrasomes have been observed *in vivo*. For example, robust migrasome formation was observed during zebrafish gastrulation (Jiang et al., 2019). Moreover, in mouse blood vessels, the real-time process of migrasome formation from neutrophils and from circulating tumor cells was observed by an intravital imaging system (Jiao et al., 2021; Wu et al., 2021). Using a set of proteins which are enriched on migrasomes but not on exosomes, migrasomes have been biochemically detected and then isolated from human serum (Zhao et al., 2019). Moreover, migrasomes have been observed in human ischemic stroke brain specimens (Schmidt-Pogoda et al., 2018). Collectively, these observations suggest that migrasomes are evolutionarily conserved organelles which form in a wide range of physiopathological settings.

## 2 Functions of migrasome

**Fig. 2:**
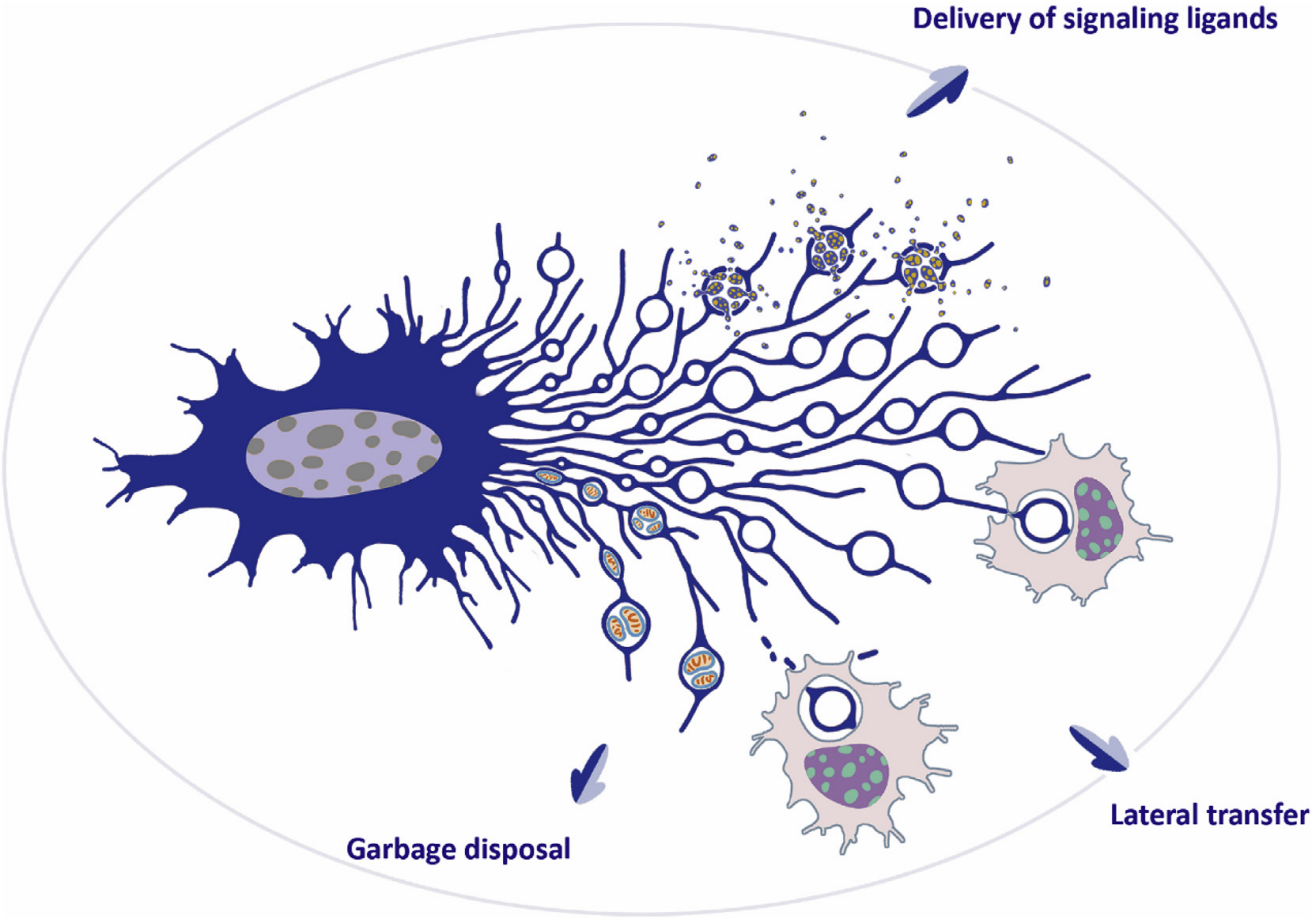
**Biological functions of migrasomes.** Biological functions of migrasomes, classified into three modes of action: (1) delivering signaling ligands to a spatially defined location, (2) disposal of garbage from the cell, and (3) mediating the transfer of biological molecules.

The biological functions of migrasomes can be summarized in terms of three modes of action: delivery of signaling ligands to a spatially defined location, disposal of garbage from cells, and transfer of biological molecules (Fig. 2). First, migrasomes act as packets of information which can be delivered to a spatially defined location to signal to the surrounding cells. In zebrafish embryonic development, proper chemotaxis of DFCs (dorsal forerunner cells) is mediated by Cxcl12 released by migrasomes. These migrasomes are generated by mesendodermal cells during gastrulation. After detaching from the parent cell, the migrasomes are concentrated in a cavity underneath the embryonic shield, where they release signaling molecules which work as regional cues to shape organ morphogenesis (Jiang et al., 2019). Second, migrasomes act as a garbage disposal mechanism by which damaged organelles are evicted from cells. Migrating cells can throw out damaged mitochondria via a migrasome-mediated process named as mitocytosis, which is important for mitochondrial quality control. Mitocytosis is crucial for maintaining neutrophil mitochondrial membrane potential and viability during circulation *in vivo* (Jiao et al., 2021). Third, migrasomes can mediate the lateral or horizontal transfer of RNAs and proteins. Migrasomes contain proteins, nucleic acids, lipids and other biomolecules. When migrasomes are engulfed by another cell, these biomolecules are transferred into the recipient cell. Recently, it was found that a set of full-length, translationally competent mRNAs are enriched in migrasomes. Once taken up by recipient cells, these migrasome-enriched mRNAs can escape from the endocytic pathway and be translated into proteins, thus modifying the recipient cell (Zhu et al., 2021).

## 3 Biogenesis of migrasomes

Migrasomes form on retraction fibers, which adhere to the surface on which the cells grow. Coating with fibronectin can significantly increase migrasome formation, indicating that adhesion is important for migrasome biogenesis. Fibronectin is an extracellular matrix protein, which can bind to Integrins to provide the adhesion force for cell migration. Moreover, mass spectrometry analysis revealed that Integrins α5 and β1 are highly enriched on migrasomes (Zhao et al., 2019), and immune-fluorescence staining using an antibody against the active form of Integrin showed that migrasome-enriched Integrins are in the activated ligand-binding state. Integrins play duel roles in migrasome formation, integrins on the cell body promote cell migration, while integrins on migrasome anchor the retraction fiber/migrasome on ECM. Since different Integrins bind to different ECM proteins, it is not surprising that migrasome formation is only promoted when Integrins are matched with their paired ECM protein (Wu et al., 2017). In live-cell images, small Integrin-positive puncta first appear on retraction fibers. Minutes later, Tspan4 is gradually recruited onto the puncta and migrasomes start to grow. Thus, the Integrin-positive, Tspan4-GFP negative puncta are defined as migrasome formation sites.

## 4 Tetraspanins

As mentioned above, Tetraspanin4 was identified as a migrasome marker in our screen for proteins that are enriched on migrasomes (Ma et al., 2015). Very soon, it become clear that ectopically expressed Tspan4 is not only enriched on migrasomes, but also promotes migrasome formation.

The first discovered Tetraspanin was CD9. In 1987, CD9 was identified as the cytotoxic target of acute lymphoblastic leukemia therapy (Zola et al., 1987). In the following years, a family of multi-membrane-spanning proteins, including ME491/CD63, OX-44/CD53, Co-029/Tspan8, CD37, and TAPA-1/CD81, were sequentially identified (Paterson et al., 1987; Hotta et al., 1988; Classon et al., 1989; Szala et al., 1990; Levy et al., 1991). In 1997, “Tetraspanin” was proposed as the name of this family to integrate the many confusing names which had emerged during the previous ten years (Maecker et al., 1997).

Tetraspanins are four-pass transmembrane proteins, with 33 family members in mammals. They share conserved tertiary structures, including a large extracellular loop (LEL), a small extracellular loop (SEL), four transmembrane domains, an N-terminal cytosolic domain and a C-terminal cytosolic domain. The extracellular parts are the most variable regions in Tetraspanins, especially the LEL, which is suggested to mediate homodimerization through a hydrophobic surface and protein-protein interactions. The C-terminal cytosolic domain usually includes motifs to sort and target the protein to a specific intracellular location, such as the lysosomal localization motif in Tspan7 and the late endosomal-lysosomal motif in CD63 (Rous et al., 2002; Bonifacino and Traub, 2003). Most Tetraspanins are post-translationally modified by glycosylation, palmitoylation and ubiquitination, which are crucial for further organization and function.

Tetraspanins are involved in multiple important biological processes, including motility, adhesion, invasion, membrane fusion and signal transduction. For example, CD9 is a target for platelet activation and regulates platelet function (Worthington et al., 1990; Ozaki et al., 2000). CD81 is the key to hepatitis C virus entry (Feneant et al., 2014). CD9 mediates sperm-oocyte fusion (Kaji and Kudo, 2004). CD63 is essential for exosome biogenesis (Pols and Klumperman, 2009). CD81 functions as a key sensor of external inputs to control the proliferation of beige adipocyte progenitor cells (APCs); it also participates in B cell development and activation (Kuppers, 2019; Oguri et al., 2020; Susa et al., 2021). CD151 contributes to tumor metastasis and could be a potential anti-tumor target (Sadej et al., 2014; Peng et al., 2020). Finally, Tspan7 is crucial for synapse maturation and function (Bassani et al., 2012).

## 5 Tetraspanins in migrasomes

**Fig. 3:**
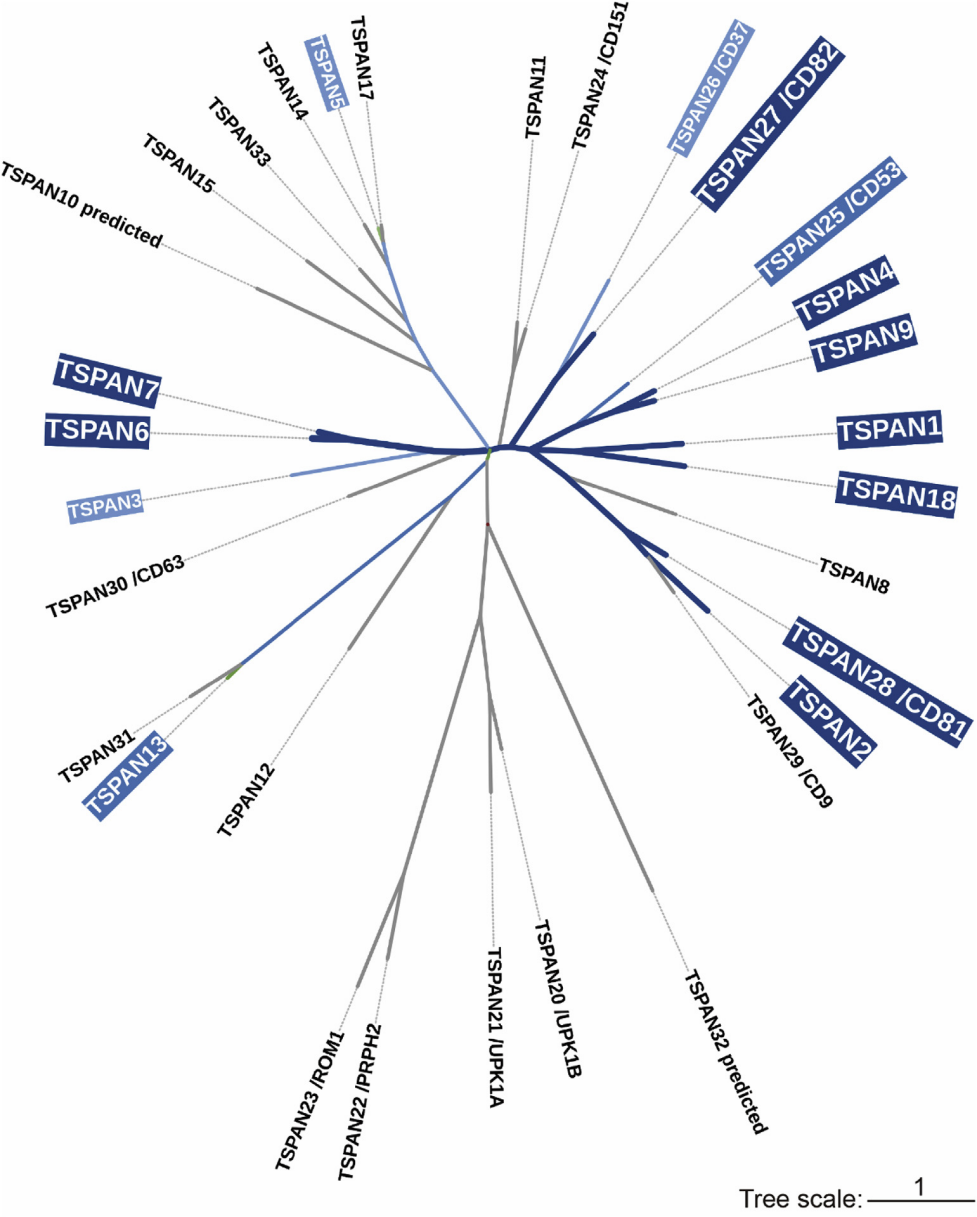
**Phylogenetic tree of Tetraspanins.** Phylogenetic unrooted tree of Tetraspanin family proteins (in Norway rat) was generated by use of MEGA11. Proteins promoting migrasome formation are highlighted in blue. The colors from dark blue to light blue represent the decreasing ability to promote migrasome biogenesis when overexpressed. Tree scale, 1.

Among the 33 family members, 14 Tetraspanin proteins can induce migrasome formation when over-expressed (Huang et al., 2019). A subset of these migrasome-promoting Tetraspanins, including Tspan4 and Tspan9, have a higher capacity for promoting migrasome formation (Huang et al., 2019; Jiao et al., 2021). By inspecting the Tetraspanin family dendrogram, we can infer that the Tspan4 branch (which includes Tspan9 and Tspan25), the Tspan1 branch, the Tspan2 branch, the Tspan27 branch and the Tspan7 branch are highly involved in promoting migrasome formation (Fig. 3). Knockout of Tspan4 in NRK and MGC803 cells significantly inhibits migrasome formation. It is worth to noting that in L929 cells, knockout of Tspan4 does not affect migrasome formation, which is likely explained by the redundancy of Tetraspanin genes (Huang et al., 2019). In zebrafish embryos, migrasome formation is significantly reduced in Tspan4 and Tspan7 knockout embryos (Jiang et al., 2019). Furthermore, in Tspan9^−/−^ mice, migrasome formation is impaired in neutrophils and bone marrow-derived macrophages (BMDMs) (Jiao et al., 2021).

The most direct evidence to support the essential role of Tetraspanins in migrasome formation comes from the *in vitro* reconstitution approach. First, we purified Tspan4 and prepared proteoliposomes with Tspan4 embedded in the membrane. Next, we generated giant unilamellar vesicles (GUVs) with or without Tspan4. We adhered the GUVs onto the bottom of a flow chamber and then used the mechanical force generated by flow to transform the vesicles into membrane tethers. This mimics the shape change which occurs on the trailing edge of a cell in response to the pulling force generated by cell migration. During the *in vitro* reconstitution process, migrasome-like structures formed during the vesicle-to-tether transformation, but only when the GUVs contained Tspan4.

To further understand how Tetraspanins participate in migrasome formation, we analyzed the Tspan4 signals during the migrasome formation process. We found that the recruitment of Tspan4 to migrasomes is correlated with the growth of migrasomes. At the early stage of migrasome biogenesis, the Tspan4 signal steadily increases on the migrasome. At the same time, the migrasome grows. Once the migrasome reaches its maximum size, the Tspan4 signal stops increasing. Based on this observation, we define the migrasome formation process as containing a growth phase and a steady phase. Using the FRAP assay, we noticed that the recruitment of Tspan4 to migrasomes is unidirectional: once recruited onto a migrasome, Tspan4 cannot move out. These data indicate that the migrasome membrane is laterally segregated from the retraction fiber membrane. Taking advantage of ultra-fast resonant scanning mode confocal imaging, we found that the Tspan4 signals, which appear to be evenly distributed on the retraction fiber when observed by a lower speed imaging mode, are actually discrete fast-moving puncta along retraction fibers and on the migrasome surface. The evenly distributed pattern observed by lower speed imaging is likely caused by motion blur, which “averages” the signal of fast-moving Tspan4 puncta along the retraction fibers. To understand the nature of these puncta, we have to go back to biophysical and biochemical studies from past decades which reveal one of the unusual properties of Tetraspanins.

## 6 Tetraspanin-enriched microdomains

Tetraspanins are able to organize a compartmentalized membrane unit by interacting with themselves, with a variety of other transmembrane and cytosolic proteins, and with a subset of lipids. These specialized membrane domains are named as Tetraspanin-enriched microdomains (TEMs). The interactions within TEMs can be divided into different levels according to the resistance to different detergents. The primary level of interaction is direct interaction between a Tetraspanin and its partner; for example, Integrins α6β1 and α3β1 directly associate with CD151 but not with other Tetraspanins (Yauch et al., 1998; Serru et al., 1999), whereas CD9P-1 and EWI-2 associate with CD9 and CD81 (Charrin et al., 2001, 2003a; Clark et al., 2001; Stipp et al., 2001a, 2001b). These direct interactions form the primary interaction blocks. Primary interaction blocks can form a dynamic network of secondary interactions through protein-protein interactions and protein-lipids interactions, which including cholesterol, gangliosides and other lipids enriched in TEMs.

Tetraspanin-enriched microdomains are enriched with a large array of transmembrane proteins including Integrins, immunoglobulin (Ig)-domain-containing factors, and receptors. In addition, Tetraspanin-enriched microdomains are enriched with a subset of lipids including cholesterol and gangliosides. Among those enriched molecules, cholesterol is the central component. Tetraspanins can bind cholesterol directly. Previous studies demonstrated that Tetraspanins such as CD9, CD81 and CD82 can physically bind to cholesterol (Charrin et al., 2003b; Silvie et al., 2006; Palor et al., 2020). The crystal structure of CD81 showed that there is a cholesterol binding pocket in the LEL domain, which can open and close to facilitate the Tetraspanin-dependent transport of CD19 to the cell surface (Zimmerman et al., 2016; Susa et al., 2021). In addition, palmitoylated Tetraspanins can interact with cholesterol through their palmitate moieties (Charrin et al., 2003b; Espenel et al., 2008; Zhu et al., 2012).

Studies of Tetraspanin-enriched microdomains were initiated mainly by biochemical approaches including detergent resistance, co-immunoprecipitation, protein cross-linking and proteomics. With the development and application of advanced detection technologies, especially super-resolution microscopy imaging, it is possible to elucidate many basic physical properties of TEMs, including their size, distribution and motility in native plasma membranes. Increasing evidence suggests that TEMs are nano-scaled assemblies, which are dispersed on cell plasma membranes (He et al., 2013; Zuidscherwoude et al., 2015; Ambrose et al., 2020). Inside TEMs, the spatial and temporal movement of the component molecules is restricted, thus maintaining the relatively high concentration of component molecules inside TEMs (Espenel et al., 2008; Yang et al., 2012). The realization that the Tspan4 puncta we observed on retraction fibers and migrasomes may be clusters of TEMs led us to the next stage of investigation.

## 7 Migrasomes are tetraspanin- and cholesterol-enriched macrodomains

Now back to migrasomes. We found that many TEM components, including Tetraspanins and Integrins are enriched on migrasomes (Wu et al., 2017; Huang et al., 2019; Zhao et al., 2019). Cholesterol, the key component for TEM formation, is highly enriched on migrasomes and its depletion impairs migrasome formation. Thus, migrasomes have a similar chemical composition to TEMs. Moreover, Tspan4 forms discrete puncta which are laterally segregated from the surrounding membrane, which suggests that these Tspan4 puncta are TEMs. The fact that Tspan4 puncta are recruited into migrasomes in a unidirectional manner during migrasome biogenesis suggests that migrasomes are formed by assembly of individual TEMs. The assembly of TEMs to form migrasomes can be observed in a modified version of the *in vitro* reconstitution system, in which the pulling force which induces the shape transformation is achieved by adhering a glass needle to the surface of the GUV, then pulling the glass needle manually by a micromanipulator. Using this method, the shape transformation process is slow, which allows us to visualize the process. Similar to the flow chamber setting, migrasome-like structures can form in the presence of cholesterol and Tspan4 when GUVs are pulled with a glass needle. Moreover, during the narrowing of the membrane tether, which is caused by the pulling force, individual Tspan4 puncta assemble into larger clusters, which then bulge out and become migrasome-like structures. This is very similar to what we observed *in vivo.* These experiments using *in vitro* systems suggest that Tspan4/cholesterol are the minimal components required for migrasome formation, and migrasome formation is driven by assembly of Tetraspanin-enriched microdomains into micrometer-scaled macrodomains, which we named as Tetraspanin-enriched macrodomains (TEMAs).

**Fig. 4:**
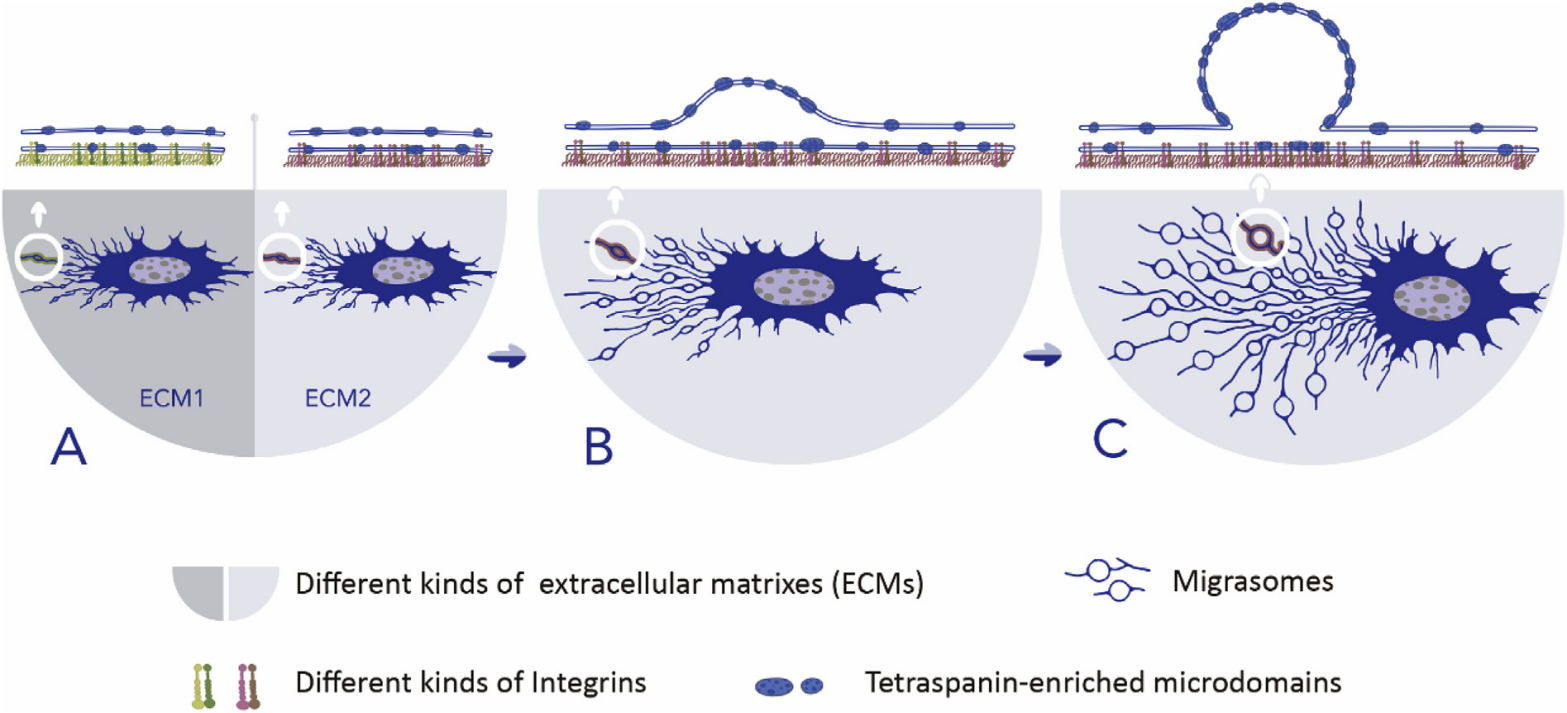
**Biogenesis of migrasomes**. (A) During cell migration, Integrins with their matched extracellular matrix proteins determine migrasome formation sites. (B) At these sites, Tspan - and cholesterol-enriched microdomains start to assemble, leading to bulge formation along the retraction fiber. (C) Finally, many TEMs assemble into micrometer-scaled macrodomains to increase the local membrane bending rigidity, which results in migrasome formation.

Finally, to understand the physical basis of the swelling of TEMAs into migrasomes, we developed a theoretical model. The key hypothesis for our model is that swelling of TEMAs is caused by the difference between the elastic properties of TEMAs and those of the retraction fiber membrane. We can understand the model by the following analogy. Imagine a rubber band, containing rigid and soft sections: when we apply a stretching force, the rigid sections will resist the thinning caused by stretching and bulge out relative to the soft sections. Computer simulation based on this model predicts that the bending rigidity of the TEMAs needs to be 5–10 fold higher than the adjacent lipid membrane to promote the swelling of TEMAs. Remarkably, this prediction was validated using atomic force microscopy to measure the bending rigidity of proteoliposomes containing increasing amounts of Tspan4 and cholesterol (Huang et al., 2019). In summary, this study revealed that migrasomes are formed by assembly of Tetraspanin-enriched microdomains into micrometer-scaled macrodomains, and the unique biophysical properties of TEMAs, combined with the pulling force generated by cell migration, gives rise to the shape transformation process which makes the migrasomes (Fig. 4).

Our study revealed that TEMs are the building blocks of migrasomes, but how are TEMs formed? What are the fundamental physicochemical principles underlying the formation of TEMs? To answer these questions, we have to understand the principles of membrane organization.

## 8 Membrane microdomains

Biological membranes provide boundaries for living cells to separate themselves from the outside environment and to create functional internal compartments. The basic structure of a membrane is the lipid bilayer, which provides two-dimensional fluidity. Amphipathic lipids, such as glycophospholipids, are the main structural lipids that form the basic matrix of a membrane bilayer. In the fluid mosaic model proposed by Singer and Nicolson in 1972, transmembrane proteins and membrane-associated proteins are randomly inlaid in or float on the lipid bilayer as mosaics and diffuse freely in ideal conditions.

Our understanding of membrane domains started in the 1960s, when researchers studied the behaviors of membranes using model membrane systems made *in vitro* using simple lipid mixtures (Heberle and Feigenson, 2011). These early studies showed that when the temperature is below the melting temperature, lipids exist in an ordered gel phase, and when the temperature is above the melting temperature, lipids transition into a disordered fluid phase. When lipids with different melting temperatures are mixed together, both gel and fluid phases can co-exist in the membrane. The next breakthrough came from the appreciation of the role of cholesterol in membranes. Cholesterol is abundant in plasma membranes. Due to the planarity of its sterol ring, cholesterol favors interactions with saturated lipids and disfavors interactions with unsaturated lipid species. This causes the lateral segregation of membranes into a cholesterol enriched, liquid-ordered phase and a liquid-disordered, more fluid phase. Sphingolipids, which favor interaction with cholesterol, are incorporated into the cholesterol-enriched, liquid-ordered phase. This observation was the key to understanding membrane microdomains.

In 1997, Simons and Ikonen proposed the “lipid rafts” hypothesis. In this hypothesis, lipid rafts — which are formed by the preferential association and lateral segregation of cholesterol, sphingolipids and specific proteins — organize membranes into different functional domains which can provide sorting and targeting signals during intracellular trafficking and signal transduction (Simons and Ikonen, 1997). The current understanding of lipid rafts is that they are dynamic nano-scaled assemblies which are enriched with cholesterol, sphingolipids and proteins such as glycosylphosphatidylinositol (GPI)-anchored proteins (Hancock, 2006). A key realization is that these assemblies of cholesterol/sphingolipids can further assemble into larger, more stable membrane domains, which is mediated by interactions between biomolecules enriched in these nano-scaled assemblies. Inter-assembly interactions, especially those mediated by protein-protein and protein-lipid interactions, give rise to a collection of membrane microdomains in an individual biological membrane system, which reflect the lipid/protein composition and the physiological state of the given membrane. Tetraspanin-enriched microdomains are one of the specialized microdomains arising from this organizational principle. These membrane domains range widely in terms of size, dynamics and temporal scale, which gives them unique chemical and biophysical properties. Moreover, since the enriched proteins vary considerably, these membrane domains can carry out a wide array of functions. They can work as structural blocks or effector modules to realize biological function, such as Tetraspanin-enriched microdomains in migrasome formation (Huang et al., 2019), and caveolae in mediating endocytosis (Anderson et al., 1992; Schnitzer et al., 1994; Murata et al., 1995). Microdomains can also act as confined reaction spaces, by creating a relatively high local concentration of all the reaction components. This greatly facilitates signal transduction. For example, organization of T cell receptor microdomains is crucial for T cell activation (Douglass and Vale, 2005). In summary, the formation of membrane domains — which is based on physiochemical properties, driven by cholesterol/sphingolipids, and facilitated by proteins — is an organizational principle which gives rise to the complex structures and functions of biological membrane.

## 9 Perspective

The realization that migrasomes are stable, micrometer-scaled membrane macrodomains made by assembling nano-scaled Tetraspanin-enriched microdomains is likely to have a profound impact on our understanding of the biology of both membrane microdomains and migrasomes. For membrane microdomains, except for a few exceptions where antibodies are used to induce large microdomains which are visible by light microscopy, there are no microdomains on unperturbed cells which are large enough and stable enough to study under the microscope. This is not a small hurdle, as interpretation of results from indirect methods for studying microdomains have caused considerable controversy which has arguably slowed the progress of the field. In this sense, migrasomes may be a perfect model system to study microdomains in a biologically relevant setting. The diameter of migrasomes is around 2 μm, which is very easy to visualize using confocal microscopy. Migrasome biogenesis takes hours, which gives us a long time window to study the assembly and dynamics of microdomains. The formation of migrasomes does not need any outside perturbation, such as antibody or ligand binding, and thus it is more physiologically relevant. Migrasomes are connected to cells by very thin retraction fibers, which means that they can be isolated from cells with high purity. This allows us to study the protein and lipid composition of membrane domains without worrying about the possible artifacts that arise from the widely used protocols to extract and isolate membrane microdomains, such as cold detergent treatment. Finally, migrasomes carry out many different functions, and some of these functions are likely dependent on the physicochemical properties of membrane microdomains. Thus, migrasomes are an ideal model system to study the functions of microdomains.

We now know that the assembly of nano-scaled TEMs is the key mechanism to drive the growth of migrasomes. Thus, insights gained from the general principles for microdomain assembly can greatly facilitate our understanding of various aspects of migrasome biology, including biogenesis and function. For example, the unique properties of TEMAs may underlie many unique behaviors of migrasomes, including the enrichment of certain cytosolic cargos in migrasomes, the leakiness of late-stage migrasomes, the enrichment of a subset of functionally important membrane proteins in migrasomes, and so on. Moreover, this understanding will translate into insight for understanding the functions of migrasomes, which will allow us to make bold new hypotheses to guide our future investigations into the physiopathological roles of migrasomes.

## Declaration of competing interest

The authors declare no conflict of interest and agree on the submission and publication of this manuscript.
