# Supplementary material for: Tetraspanin-enriched microdomains: The building blocks of migrasomes
Source: Cell Insight. 2022 Jan 5;1(1):100003. doi: 10.1016/j.cellin.2021.100003 (PMC10120322; doi:10.1016/j.cellin.2021.100003)
Supplement: Multimedia component 1 [file mmc1.docx]

**Cover image caption**

Heat-map image of migrasomes, produced by Tspan4-mCherry L929 cell (the upper one) and Tspan4-GFP L929 cell (the lower one). The image was captured by confocal microscopy.
